# Data-Driven Modeling of Smartphone-Based Electrochemiluminescence Sensor Data Using Artificial Intelligence

**DOI:** 10.3390/s20030625

**Published:** 2020-01-23

**Authors:** Elmer Ccopa Rivera, Jonathan J. Swerdlow, Rodney L. Summerscales, Padma P. Tadi Uppala, Rubens Maciel Filho, Mabio R. C. Neto, Hyun J. Kwon

**Affiliations:** 1 Department of Engineering, Andrews University, Berrien Springs, MI 49104, USA; elmeralbertocr@gmail.com (E.C.R.); mabioc@andrews.edu (M.R.C.N.); 2 School of Chemical Engineering, University of Campinas, Campinas 13083-852, Brazil; maciel@feq.unicamp.br; 3 Department of Computing, Andrews University, Berrien Springs, MI 49104, USA; swerdloj@gmail.com (J.J.S.); summersc@andrews.edu (R.L.S.); 4 School of Population Health, Nutrition & Wellness, Andrews University, Berrien Springs, MI 49104, USA; padma@andrews.edu

**Keywords:** electrochemiluminescence, artificial intelligence, sensor, mobile phone, modeling

## Abstract

Understanding relationships among multimodal data extracted from a smartphone-based electrochemiluminescence (ECL) sensor is crucial for the development of low-cost point-of-care diagnostic devices. In this work, artificial intelligence (AI) algorithms such as random forest (RF) and feedforward neural network (FNN) are used to quantitatively investigate the relationships between the concentration of $\;\mathrm{Ru}{\left(\mathrm{bpy}\right)}_{3}^{2+}$ luminophore and its experimentally measured ECL and electrochemical data. A smartphone-based ECL sensor with $\;\mathrm{Ru}{\left(\mathrm{bpy}\right)}_{3}^{2+}$/TPrA was developed using disposable screen-printed carbon electrodes. ECL images and amperograms were simultaneously obtained following 1.2-V voltage application. These multimodal data were analyzed by RF and FNN algorithms, which allowed the prediction of $\;\mathrm{Ru}{\left(\mathrm{bpy}\right)}_{3}^{2+}$ concentration using multiple key features. High correlation (0.99 and 0.96 for RF and FNN, respectively) between actual and predicted values was achieved in the detection range between 0.02 µM and 2.5 µM. The AI approaches using RF and FNN were capable of directly inferring the concentration of $\;\mathrm{Ru}{\left(\mathrm{bpy}\right)}_{3}^{2+}$ using easily observable key features. The results demonstrate that data-driven AI algorithms are effective in analyzing the multimodal ECL sensor data. Therefore, these AI algorithms can be an essential part of the modeling arsenal with successful application in ECL sensor data modeling.

## 1. Introduction

Electrochemiluminescence (ECL) is being explored in research ranging from fundamental studies to its application as a platform of light-emitting sensors and an analytical detection method. Because ECL does not requires any external excitation light source, it has the advantage of having ultra-sensitivity and very low background signal. In addition, it allows minimal instrumentation due to the simplicity of voltage application, rapid measurements (only a few seconds), localized light emission (geometric location of light on a working electrode), and cost-effective set-up [1]. These are the inherent advantages of ECL over other light emission-based techniques such as photoluminescence and chemiluminescence [2]. In this context, the smartphone can be an alternative to the expensive traditional instrumentation for ECL sensors such as the photomultiplier tube (PMT). Smartphones are typically equipped with powerful data transmission capabilities and have powerful processors for storage and analysis of imaging data. Recent literature shows that the use of smartphones toward optical biosensing is particularly important in the study of health [3], security [4], and environment [5].

Recent research is focused on the development of instrumentation with adequate electrochemical and chemiluminescent functionality to achieve reproducibility [6]. Meanwhile, the optimization of the ECL performance, which is closely related to the increase in signal intensity, is being addressed through the design of novel luminophores and coreactants, as well as the development of assay-driven strategies using existing luminophores and coreactants [7,8,9]. Tris (2, 2′-bipyridine) ruthenium(II) ($\mathrm{Ru}{\left(\mathrm{bpy}\right)}_{3}^{2+}$) with tripropylamine (TPrA) as a coreactant is one of the most widely studied ECL systems; however, its reactions are not understood clearly so far due to its multiparametric nonlinear nature [9,10].

Quantitative studies to explore the complex mechanism of ECL typically use applied mathematical methods, particularly partial differential equations (PDEs) that constitute mechanistic or first-principle models. This modeling approach is suitable for a certain class of problems that are susceptible to a mathematical description such as the $\mathrm{Ru}{\left(\mathrm{bpy}\right)}_{3}^{2+}$/TPrA system charge, momentum, and mass transfer, as well as the reaction rates involved. Most of these studies use the commercial software COMSOL Multiphysics^®^ that, through the finite element method, solves the constituent PDEs [11]. Among them, the studies of Danis et al. [6,7], which used mechanistic models combined with spectroelectrochemistry, effectively predict the concentration of luminophore and ECL emission. In other work [12], model simulations coupled to microscopy imaging provided light emission mechanism insight to obtain high sensitivity in bead-based ECL assays. These studies required strong expertise in electrochemical theory for the mechanistic model set-up. In this respect, the emergence of easy-to-use software such as KISSA [13] could significantly bring down the barriers to modeling electrochemical phenomena. As an example, this software was used to study the effect of the diffusion rates of reactants on ECL emission for the $\mathrm{Ru}{\left(\mathrm{bpy}\right)}_{3}^{2+}$/TPrA system with reduced computational cost as compared to commercial software [14].

As previously discussed, the laws of conservation of charge, momentum, and mass are currently carried out without requiring expert knowledge of numerical analysis. The real challenge is defining appropriate mathematical representation of reaction rates and estimating their kinetic parameters. As ECL analysis is strongly dependent on the sensing conditions, any changes in these conditions also have a significant impact on the values of the kinetic parameters. Even if the reaction rates are applicable, a re-estimation of the kinetic parameters is required under different conditions. For this, it is necessary to obtain the experimental measurements of the main state variables (e.g., concentration of luminophore and co-reactant) over the course of ECL reaction at regular time intervals, which is not a straightforward task [6]. The proper choice of the reaction rates and their corresponding kinetic parameters to propose a reliable mechanistic model is the subject of considerable discussion in recent literature [6,7,15,16]. In other approaches, the so-called calibration curve, i.e., a regression equation, can be useful to infer the concentration of $\mathrm{Ru}{\left(\mathrm{bpy}\right)}_{3}^{2+}$ if it is correlated with a key feature of the system such as the maximum value of the ECL intensity. Nevertheless, this approach is oversimplified because it requires the predetermination of a single key feature that may not have sufficient information of the system, and it also requires a recalibration for different sensing conditions.

As an alternative to the mechanistic approach and regression equations, the use of data-driven models supported by artificial intelligence (AI) is becoming an essential part of the modeling arsenal with successful applications in many fields [17]. However, to the best of the authors’ knowledge, there is no literature on ECL system modeling using AI algorithms. These algorithms, such as neural networks and random forest, greatly improved the predictive accuracy of data regression [18]. AI algorithms can combine several sources of multimodal data into a single, predictive AI-based model, providing maximum approximation of the phenomenon without the complexity and uncertainty. AI enables the use of variables that could not be included in the mechanistic model due to a lack of understanding [19,20].

**Figure 1:**
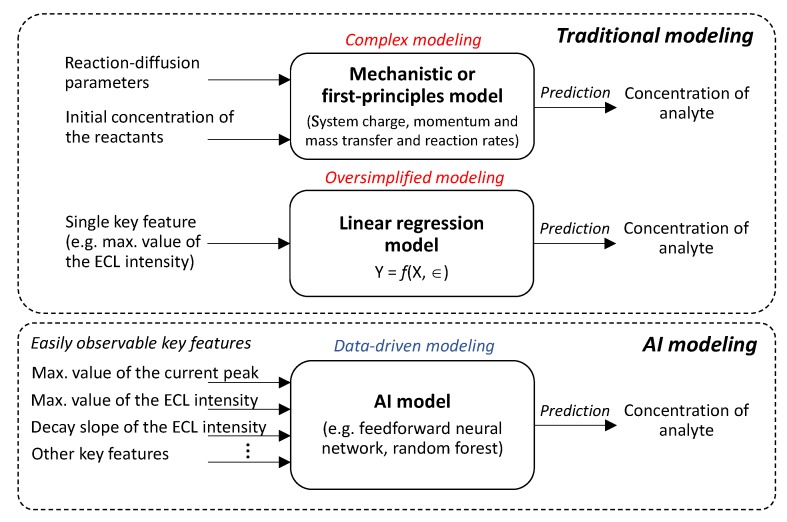
Schematic diagram of the comparison of the traditional and artificial intelligence (AI) modeling in the estimation of the analyte concentration.

This study investigated the quantitative cause-and-effect relationships between the concentration of $\mathrm{Ru}{\left(\mathrm{bpy}\right)}_{3}^{2+}$ luminophore and its experimentally measured ECL and electrochemical features. A data-driven model supported by AI algorithms was able to predict the luminophore concentration from easily measurable features obtained from sequences of ECL imaging and amperograms. The performance of the AI algorithms, namely, random forest (RF) and feedforward neural network (FNN), was compared in terms of performance measurements to assess the predictive capability of each algorithm. Figure 1 summarizes the comparison of the traditional modeling and the proposed modeling in the estimation of the analyte concentration.

## 2. Materials and Methods

### 2.1. Chemical and Reagents

All experiments were conducted using tris (2,2′-bipyridyl) dichlororuthenium (II) hexahydrate (Ru(bpy)_3_Cl_2_∙6H_2_O) and a coreactant of tri-*n*-propylamine (TPrA) purchased from Sigma Aldrich (now Millipore Sigma, St. Louis, MO, USA). The supporting electrolyte phosphate buffer solutions (PBS) were prepared by dissolving PBS tablets (Sigma Aldrich, St. Louis, MO, USA) in water (pH 7.4). All aqueous solutions were prepared with Milli-Q water purchased from APS Water Services Corp., Van Nuys, CA, USA (resistivity ≥ 18.2 MΩ⸱cm).

### 2.2. Sensor Apparatus and Electrodes

**Figure 2:**
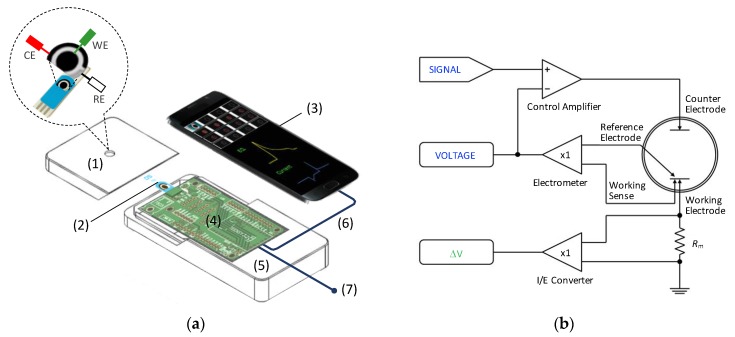
Schematic diagram of (**a**) the mobile phone-based electrochemiluminescence (ECL) sensor apparatus that mainly comprises (1) a magnifying lens, (2) screen-printed electrodes, (3) a smartphone, (4) a potentiostat circuit, (5) a light-tight container, (6) a Universal Serial Bus (USB) cable, and (7) a cable to the battery or USB port; (**b**) the basic operation of the portable potentiostat circuit.

Simultaneous measurements of sequences of ECL imaging and amperograms (current vs. time) were carried out using a mobile phone-based ECL sensor apparatus. The sensor design interfaces with a custom compact potentiostat and a mobile phone (Samsung Galaxy S7) with a custom-made app controlling the potentiostat parameters and the phone camera for time synchronization (Figure 2a). The compact potentiostat used was customized from an open-source potentiostat shield named Rodeostat (designed from the Teensy 3.2 board; IO Rodeo, Pasadena, CA, USA) in a three-electrode set-up. Disposable screen-printed carbon electrodes (DropSens, DRP-110) were used consisting of a carbon working electrode (4 mm diameter), a carbon ink counter electrode, and a silver reference electrode printed on a flat ceramic card. Figure 2b illustrates the basic operation of the portable potentiostat circuit. The signal and the voltage (in blue letters) are generated through the microcontroller unit (MCU) attached on the board. The MCU is modulated according to a square waveform signal (however, it could also be a sine or triangular waveform) and an input voltage. The signal and the voltage feed the control amplifier, which is a servo amplifier, to adjust the amplitude to the desired current applied on the counter electrode. During tests, the electrometer measures the voltage differences between the reference and working electrodes and retro-feeds the control amplifier to keep the voltage at the desired value. The current flowing through the working electrode is measured at the I/E converter, which is a current-to-voltage converter, and it is recorded and displayed as a current vs. time graph. The phone camera was set to pro mode with autofocus mode at ISO 3200, and burst mode was used to collect two-dimensional (2D) ECL image sequences with 8–20 frames per second (FPS). During experiments, the cell phone camera was aligned with the hole of the container to fit the mobile phone camera and placed just above the working electrode. The custom potentiostat was connected with the cell phone on one side and the screen-printed electrodes (SPEs) on the other side.

### 2.3. Assays

A 1 mM stock solution of $\mathrm{Ru}{\left(\mathrm{bpy}\right)}_{3}^{2+}$ in Milli-Q water was diluted to provide sample solutions from 0.02 to 2.5 µM of $\mathrm{Ru}{\left(\mathrm{bpy}\right)}_{3}^{2+}$. Each sample solution was mixed with 20 mM TPrA in 0.1 M PBS, constituting a $\mathrm{Ru}{\left(\mathrm{bpy}\right)}_{3}^{2+}$/TPrA system. The reproducibility and repeatability assessment of this system was demonstrated elsewhere [1]. Measurements were performed at room temperature by dropping 50 µL of $\mathrm{Ru}{\left(\mathrm{bpy}\right)}_{3}^{2+}$/TPrA solution onto the carbon working electrode surface. A waiting time of 10 min was established to create less electrode contact resistance. Then, the ECL reaction was triggered by applying 1.2 V, while simultaneously measuring the ECL emission and the current at the carbon working electrode.

### 2.4. Electrochemical and ECL Experimental Data Generation

Experimental data generation is a critical step in the construction of AI algorithms. The performance of the AI algorithms depends largely on the quality of the data used in the training step. This study used electrochemical and ECL data from measurements performed with the mobile phone-based ECL sensor for training the AI algorithms.

**Figure 3:**
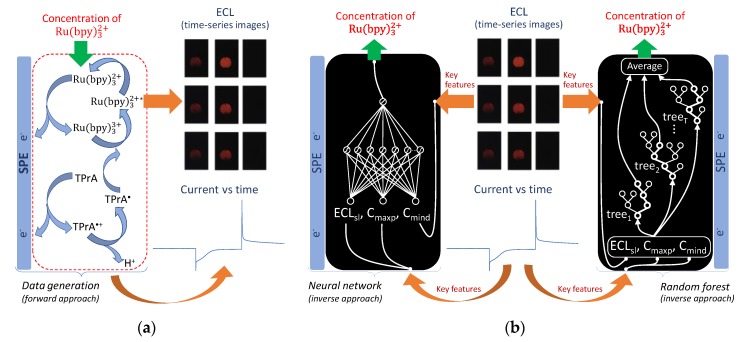
Schematic diagrams of (**a**) the procedure for experimental data generation (forward approach) using the mobile phone-based ECL sensor and (**b**) data-driven modeling (inverse approach) using a feedforward neural network and a random forest.

**Figure 4:**
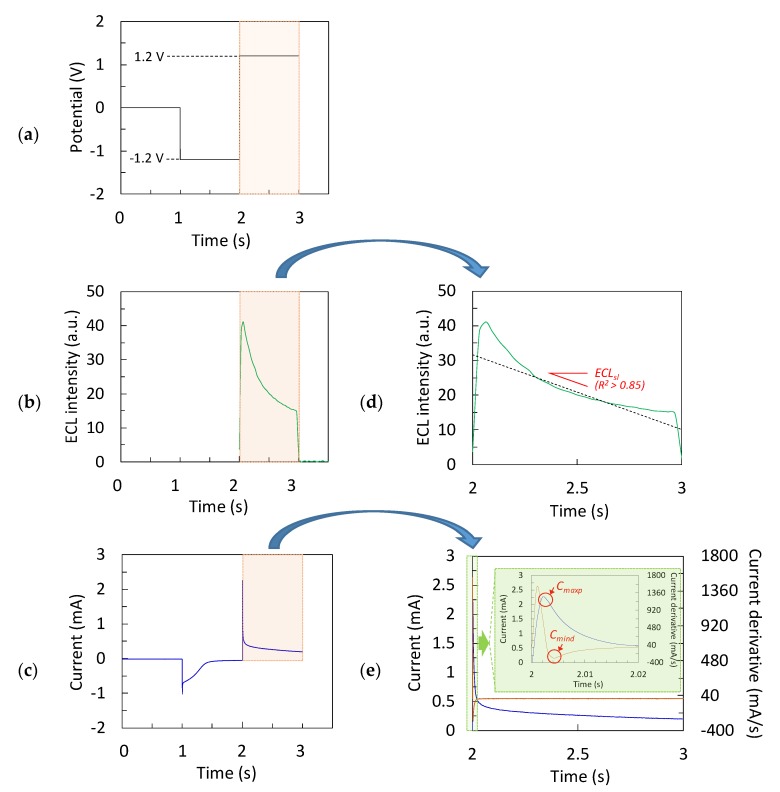
Chronoamperometry technique: (**a**) potential vs. time applied on carbon working electrode, (**b**) typical ECL response vs. time (green line), (**c**) typical current response vs. time (blue line), (**d**) zoom-in view of the shaded red box in Figure 4b, (**e**) zoom-in view of the shaded red box in Figure 4**c**. Figure 4e also shows the current derivative signal (brown line) corresponding to the current response; the green box magnifies these responses. C_maxp_: maximum value of the current peak, C_mind_: minimum derivative value of the current, ECL_sl_: decay slope of the ECL intensity.

The procedure for experimental data generation used a forward approach as illustrated in Figure 3a, where the electrochemical and ECL data were determined given a concentration of $\mathrm{Ru}{\left(\mathrm{bpy}\right)}_{3}^{2+}$. In this procedure, the ECL sensor explored the chronoamperometry technique (an example of real data is shown in Figure 4), where a square waveform potential was applied to the carbon working electrode with 50 µL of $\mathrm{Ru}{\left(\mathrm{bpy}\right)}_{3}^{2+}$/TPrA sample solution. To simultaneously measure the electrochemical and ECL data for each concentration of $\mathrm{Ru}{\left(\mathrm{bpy}\right)}_{3}^{2+}$, the portable potentiostat was set to apply a potential of 0 V vs. Ag/Ag+ for 1 s, followed by −1.2 V vs. Ag/Ag+ for 1 s, and finally followed by 1.2 V vs. Ag/Ag+ for 1 s (Figure 4a). The potentials 0 V vs. Ag/Ag^+^ and −1.2 V vs. Ag/Ag^+^ were used to stabilize the system while avoiding oxidation of $\mathrm{Ru}{\left(\mathrm{bpy}\right)}_{3}^{2+}$. The potential of 1.2 V vs. Ag/Ag^+^ produced ECL upon concomitant oxidation of $\mathrm{Ru}{\left(\mathrm{bpy}\right)}_{3}^{2+}$ and TPrA. Typical transient current and ECL responses recorded over the course of the stabilization and oxidation periods are shown in Figure 4b,c, respectively. Figure 4d,e show the zoom-in view of the shaded area in Figure 4b,c, respectively. Figure 4e also shows the current derivative signal (brown line) corresponding to the current response (blue line). From this data, three key features were identified: the maximum value of the current peak (C_maxp_), the minimum derivative value of the current (C_mind_), and the decay slope of the ECL intensity (ECL_sl_), shown in red letters (Figure 4d,e). It is worth mentioning that the estimated slopes explained the decay of ECL intensities accurately with a coefficient of determination, *R^2^*, above 0.85 for all measurements. The three key features chosen were the input variables of the data-driven models. The output variable was the concentration of $\mathrm{Ru}{\left(\mathrm{bpy}\right)}_{3}^{2+}$.

Following the procedure described above, multiple experiments were performed for different concentrations of $\mathrm{Ru}{\left(\mathrm{bpy}\right)}_{3}^{2+}$ distributed in a range of 0.02 to 2.5 µM. This range was established based on prior knowledge of the ECL emission for the $\mathrm{Ru}{\left(\mathrm{bpy}\right)}_{3}^{2+}$/TPrA system [1]. Experimental profiles for C_maxp_, C_mind_, and ECL_sl_ were thereby obtained as a function of concentration of $\mathrm{Ru}{\left(\mathrm{bpy}\right)}_{3}^{2+}$. The goal was to include the data containing the most relevant information about the system in the training data. A routine implemented in the R programming environment was used to interpolate these measurements that showed consistent trends in order to increase the dataset. Therefore, the dataset used for training provided 105 interpolated data points for each input variable and the same amount of data for the corresponding output variable.

The modeling supported by AI algorithms used an inverse approach unlike the forward approach used for data generation (and also used in the mechanistic modeling), as shown in Figure 3b. In the inverse approach, the data-driven model is considered as a black-box model that learns to relate the inputs, C_maxp_, C_mind_, and ECL_sl_ to the output, i.e., the concentration of $\mathrm{Ru}{\left(\mathrm{bpy}\right)}_{3}^{2+}$, from a large number of sample points. Due to the models supported by AI having very limited extrapolation properties, their predictions are only valid when using values within the range defined by the limits for the input variables.

### 2.5. AI algorithms

#### 2.5.1. Random Forest (RF)

A random forest algorithm is a widely used nonparametric technique for data classification and regression analysis. A detailed description of the fundamentals of RF is given by Breiman [21]. In this study, the focus is on the application of RF to obtain a regression between the input variables (C_maxp_, C_mind_, and ECL_sl_) and an output variable (concentration of $\mathrm{Ru}{\left(\mathrm{bpy}\right)}_{3}^{2+}$). The idea of RF is to construct a set of trees from samples randomly selected from the training set by a bootstrapping technique and to generate an average prediction of the individual trees. Overfitting is avoided by the division of nodes into decision trees where the RF algorithm randomly selects a subset of variables for each node. The average of the values in the terminal nodes of the decision trees was used to estimate the concentration of $\mathrm{Ru}{\left(\mathrm{bpy}\right)}_{3}^{2+}$ (Figure 3b). Therefore, the predicted value by the entire random forest, *h_j_*, is denoted by Equation (1).

(1) $$h_{j}\;=\;\sum_{t=1}^{T}h_{jt},\;(t\;=\;1,\;\ldots ,\;T)\;\mathrm{and}\;(j\;=\;1,\;\ldots ,\;n_{sample}),$$

where *h_jt_* represents the predicted value concentration of $\mathrm{Ru}{\left(\mathrm{bpy}\right)}_{3}^{2+}$ by tree *t*, *T* represents the total number of trees, and *n_sample_* represents the total number of samples from training set.

The leave-one-out cross-validation (LOOCV) technique was employed to train the RF algorithm. In LOOCV, n − 1 samples from the training set are used to train the RF, and the remaining sample is used to evaluate the accuracy; this was repeated 90 times. The RF tuning parameters for the LOOCV were the number of trees to be grown (n_tree_), the number of predictor variables used to split the nodes at each partitioning (mtry), and the minimum size of the terminal node or leaf (node size). RF accuracy was assessed on the validation and testing set using performance measures such as mean square error (MSE) and the coefficient of determination (*R^2^*). The RF was implemented in the R programming environment using the randomForest package Version 4.6-14 [22], based on Breiman and Cutler’s Fortran code [21].

#### 2.5.2. Feedforward Neural Network (FNN)

This work uses an FNN-type artificial neural network (ANN) [23] due to its simple mathematical form and logical architecture for data-driven modeling. These characteristics make it suitable for implementation in a prediction framework, where reduced mathematical complexity is an important factor for real-time prediction. The FNN with an input layer, one hidden layer of sigmoidal neurons, and a layer of linear output neurons was used in this study, where the numbers of neurons were *I*, *J*, and *M*, respectively. The neurons are highly interconnected by weights and bias parameters. Mathematically, the FNN can be represented as Equation (2).

(2) $$g_{m}=F\left(\sum_{j=1}^{J}W_{mj}f\left(\sum_{i=1}^{I}w_{ji}x_{i}+\theta_{j}\right)+b_{m}\right),\;(j\;=\;1,\;\ldots ,\;J),\;(i\;=\;1,\;\ldots ,\;I)\;\mathrm{and}\;(m\;=\;1,\;\ldots ,\;M),$$

where *g_m_* and *x_i_* represent the vector of input and output variables, *f*(∙) and *F*(∙) represent the activation functions of the *j-*th neuron in the hidden layer and of the *m-*th neuron in the output layer, respectively, *w_ji_* denotes the weight connecting the *i-*th neuron in the input layer and the *j-*th neuron in the hidden layer, *θ_j_* denotes the bias of the *j-*th neuron in the hidden layer, *W_mj_* denotes the weight connecting the *j-*th neuron in the hidden layer and the *m-*th neuron in the output layer, and *b_m_* denotes the bias in the *m-*th neuron in the output layer.

Figure 3b details the input variables (C_maxp_, C_mind_, and ECL_sl_) and the output variable (concentration of $\mathrm{Ru}{\left(\mathrm{bpy}\right)}_{3}^{2+}$) used to perform the FNN training. A representative dataset comprising 105 input/output samples was presented to the FNN for estimating the weight and bias (FNN parameters). The data were randomly divided into a training set and a validation set. The predictive performance of FNN was assessed using different measurements (testing set) performed with the mobile phone-based ECL sensor. The appropriate number of neurons in the hidden layer that prevents overfitting of the model and achieves a good generalization of training was determined by cross-validation (CV). CV means that FNNs with different numbers of hidden neurons, that is, different architectures, are trained with the training set, and the performances are assessed on the ability to make accurate predictions of the validation set in terms of *R^2^* and MSE. The FNN was implemented in the R programming environment using the neuralnet package Version 1.44.2 [24].

## 3. Results and Discussion

### 3.1. Chronoamperometric Data for Data-Driven Modeling

**Figure 5:**
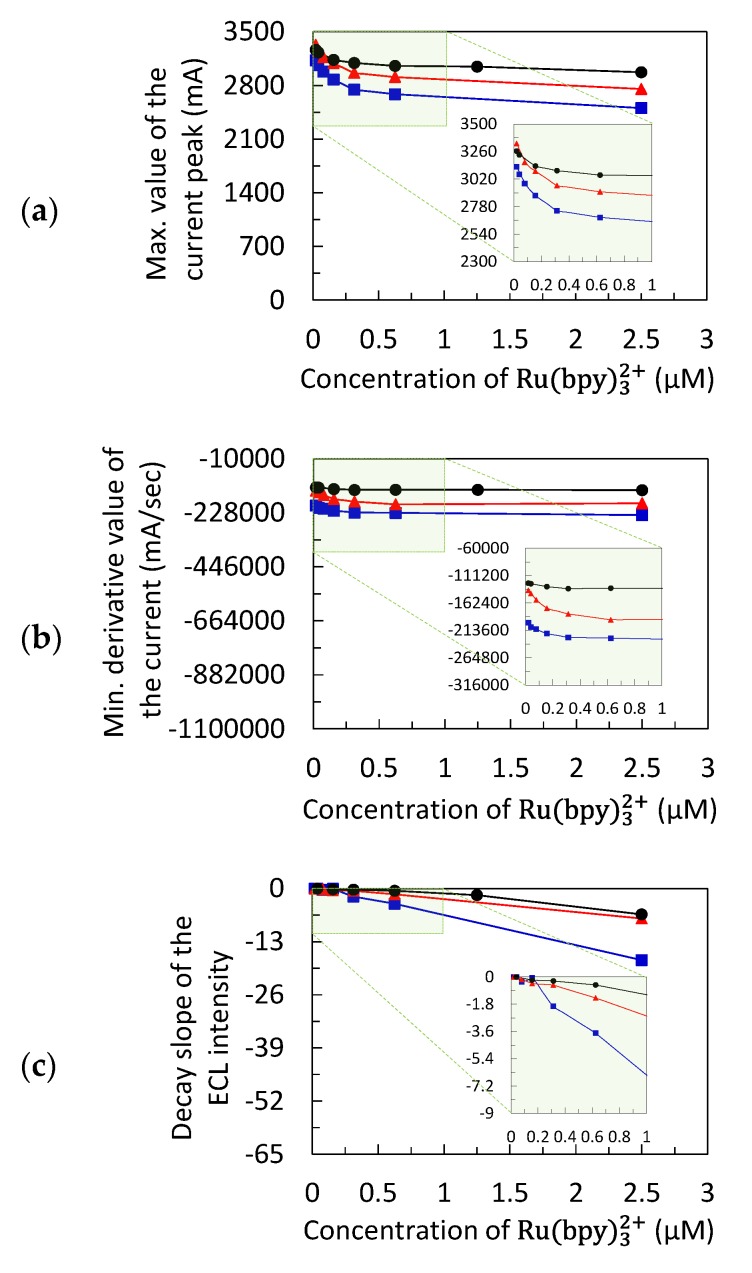
Measurements (black, red, and blue solid symbols are for repetitions 1, 2, and 3, respectively) and interpolated data (continuous lines) used to train the random forest (RF) algorithm: (**a**) maximum value of the current peak, C_maxp_, (**b**) minimum derivative value of the current, C_mind_, and (**c**) decay slope of the ECL intensity, ECL_sl_.

A series of chronoamperometric measurements were performed using the mobile phone-based ECL sensor. The ECL and electrochemical key features were measured at different concentrations of $\mathrm{Ru}{\left(\mathrm{bpy}\right)}_{3}^{2+}$ (from 0.02 to 2.5 µM) following the approach proposed in Section 2.4. The key features identified were the maximum value of current peak, C_maxp_, the minimum derivative value of the current, C_mind_, and the decay slope of the ECL intensity, ECL_sl_. The concentrations of $\mathrm{Ru}{\left(\mathrm{bpy}\right)}_{3}^{2+}$ were consistent with the practical use of this luminophore as a label. Figure 5 shows the behavior of each key feature considered in this study as a function of the concentration of $\mathrm{Ru}{\left(\mathrm{bpy}\right)}_{3}^{2+}$. These data clearly demonstrate the influence of the concentration of the luminophore on C_maxp_, C_mind_, and ECL_sl_. As concentration of $\mathrm{Ru}{\left(\mathrm{bpy}\right)}_{3}^{2+}$ increased from 0.02 to 2.5 µM, the key electrochemical features, C_maxp_ and C_mind_, decreased as shown in Figure 5a,b, respectively. Meanwhile, ECL_sl_ exhibited lower values at higher concentration of $\mathrm{Ru}{\left(\mathrm{bpy}\right)}_{3}^{2+}$ (Figure 5c). Previous studies [7,25] discussed the importance of having systems capable of performing ECL and electrochemical measurements in sync to develop models that investigate the mechanism of the $\mathrm{Ru}{\left(\mathrm{bpy}\right)}_{3}^{2+}$/TPrA system. The consistent downward trend of experimental measurements of C_maxp_, C_mind_, and ECL_sl_ with the concentration of the luminophore made it possible for these measurements to be interpolated to generate a large dataset. This strategy allowed for well-distributed data of the key features for the calibration of the AI algorithms. This is a very critical issue that should be addressed, as AI algorithms have very limited extrapolation properties [26]. For example, Figure 5a–c show the measurements (solid symbols) and the interpolated data (continuous lines) used to calibrate the random forest (RF) algorithm. These data and those for calibration of the feedforward neural network (FNN) were randomly divided into a training set (85%) and a validation set (15%). Prior to interpolation, three experimental measurements (i.e., three amperograms and three sets of ECL images) were randomly extracted from the original set of experimental measurements, which determined the testing set.

### 3.2. Data-Driven Model Calibration and Prediction of $Ru{\left(bpy\right)}_{3}^{2+}$

#### 3.2.1. Random Forest (RF) Prediction Results

**Figure 6:**
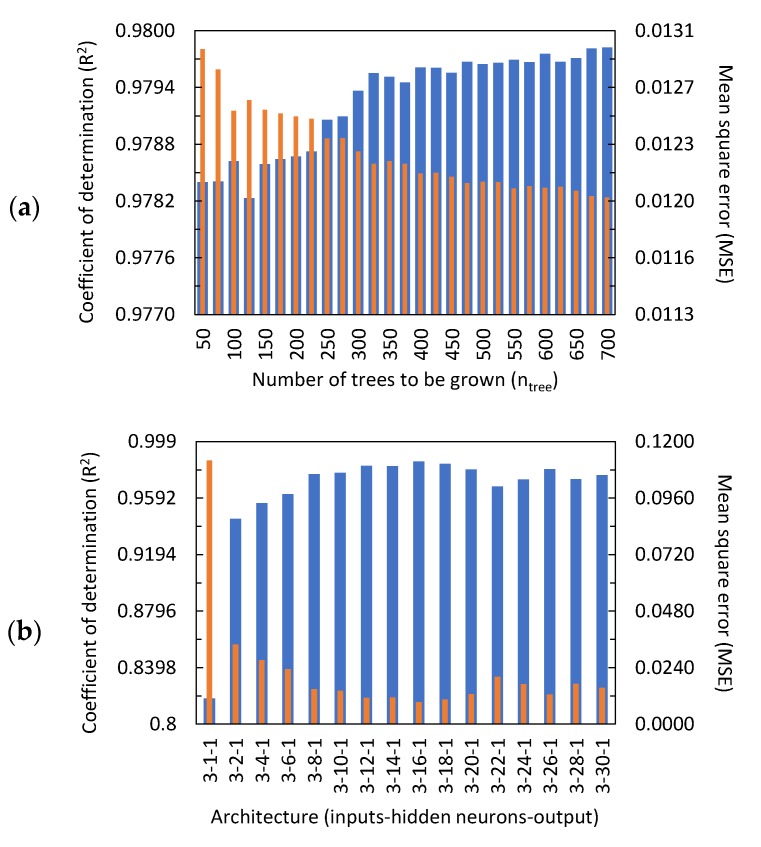
Performance measures (*R^2^* and mean square error (MSE)) to evaluate the accuracy of (**a**) the random forest (RF) at different random of trees to be grown (n_tree_) and (**b**) the feedforward neural network (FNN) at different architectures (inputs-hidden neurons-output). Blue bars represent *R^2^* (left axis), and orange bars represent MSE (right axis).

**Figure 7:**
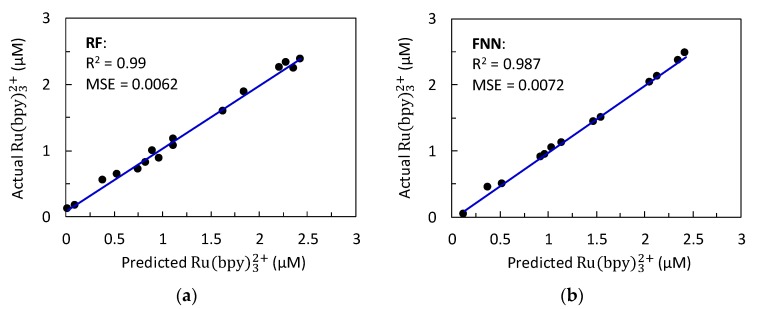
Actual versus predicted values of the concentration of $\mathrm{Ru}{\left(\mathrm{bpy}\right)}_{3}^{2+}$ obtained for validation set using (**a**) random forest and (**b**) feedforward neural network.

Several structures of the random forest (RF) with different n_tree_ (number of trees to be grown) were compared to build the model based on RF. The model estimates the concentration of $\mathrm{Ru}{\left(\mathrm{bpy}\right)}_{3}^{2+}$ using the maximum value of the current peak, C_maxp_, the minimum derivative value of the current, C_mind_, and the decay slope of the ECL intensity, ECL_sl_, as input variables. Figure 6a shows that, at values greater than n_tree_ of 500, the MSE and R^2^ did not show significant improvement. Therefore, the RF tuning parameter, n_tree_, for the leave-one-out cross-validation (LOOCV) technique was determined to be 500. The remaining tuning parameters were fixed as follows [22]: number of predictor variables used to split the nodes at each partitioning (mtry) = 1.732 (square root of the number of inputs), and minimum size of the terminal node or leaf (node size) = 5. The accuracy of the generated model by the LOOCV technique was assessed by predicting the concentration of $\mathrm{Ru}{\left(\mathrm{bpy}\right)}_{3}^{2+}$ for the validation set. Figure 7a shows the actual versus predicted values for this set. The corresponding assessment using the performance measures, *R^2^* and MSE, demonstrated that the model predictions were particularly accurate. As for the testing set, the RF prediction results were similar to those observed for the validation set. The actual versus predicted values and the performance measures are presented in Table 1. The results showed that the model based on RF can effectively directly infer the concentration of the $\mathrm{Ru}{\left(\mathrm{bpy}\right)}_{3}^{2+}$ from certain key features from multimodal data of the mobile phone-based ECL sensor. To the best of the authors’ knowledge, the RF was not previously used for the regression analysis of data from electrochemical/ECL sensors because it is relatively easier to understand the mathematical form of parametric models such as the FNN. RF can achieve high precision when a large number of input variables with a large amount of data are used [27]. Nevertheless, this study shows that the use of a reduced number of significant input variables (called key features) achieves accurate prediction results. These results were slightly higher than those found using FNN, as shown in the next section.

#### 3.2.2. Feedforward Neural Network (FNN) Prediction Results

Different network architectures with a single hidden layer were compared to build the data-driven model based on an FNN that predicts the concentration of $\mathrm{Ru}{\left(\mathrm{bpy}\right)}_{3}^{2+}$. The optimal architecture was determined by varying the number of neurons in the hidden layer. In total, 16 architectures were assessed as shown in Figure 6b. The appropriate number of neurons in the hidden layer was chosen using cross-validation with the number of training epochs fixed at 1.0 × 10^5^ for all the architectures studied. The FNN with 16 hidden neurons was determined to give the lowest MSE and *R^2^* closer to that for the validation set (Figure 6b). Thus, the optimized model used a 3-16-1 (input-hidden neurons-output) architecture containing 81 parameters (weights and bias). Table 2 shows the FNN optimized parameters according to the notation of Equation (2). The comparison between the actual values of the concentration of $\mathrm{Ru}{\left(\mathrm{bpy}\right)}_{3}^{2+}$ and the corresponding predicted values by the optimized model for the validation set is shown in Figure 7b. The results showed that the model accurately predicted the concentration of $\mathrm{Ru}{\left(\mathrm{bpy}\right)}_{3}^{2+}$, as assessed by the *R^2^* and MSE. For the testing set, it can be seen from Table 1 that the model based on the FNN also described the experimental measurements accurately (*R^2^* = 0.961, MSE = 0.0356). Nevertheless, the accuracy of this prediction was slightly lower than that observed using random forest (*R^2^* = 0.996, MSE = 0.0012). Previous studies [28,29] showed that the use of FNN as a data regression method in the development of sensors based on electrochemical measurements provided prediction results with high precision. However, to the best of the authors’ knowledge, this is the first study to predict the concentration of a compound using key features from multimodal data (ECL imaging and amperograms) into a single FNN. While FNNs achieved acceptable prediction accuracy for the testing set in this study, further investigations could be performed using deep learning to improve the prediction accuracy of the neural networks. Recent advances in training techniques and increased computational resources made it possible to construct deep neural networks such as the convolutional neural network [30] and recurrent neural network [31]. These novel architectures could be applied to the development of the ECL sensors as they are particularly useful for image processing and time series data.

#### 3.2.3. Visualizing Relationships between the Key Features and the Concentration of $\mathrm{Ru}{\left(\mathrm{bpy}\right)}_{3}^{2+}$

**Figure 8:**
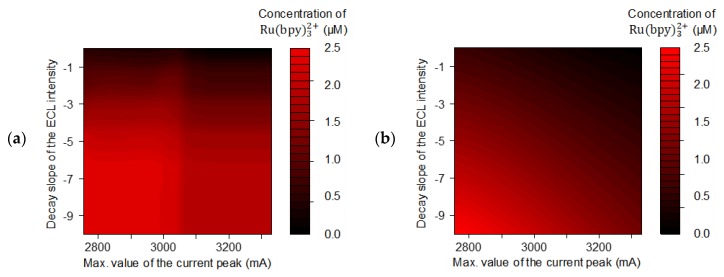
Contour plot generated by (**a**) random forest and (**b**) feedforward neural network at a fixed C_mind_.

Contour plots were generated from the validated models (Figure 8a,b for RF and FNN, respectively) for the visualization of the relationships between the input variables (C_maxp_ and ECL_sl_) and the concentration of $\mathrm{Ru}{\left(\mathrm{bpy}\right)}_{3}^{2+}$ (response variable). It can be seen that the contours for both the FNN and the RF were nonlinear and revealed that the concentration of $\mathrm{Ru}{\left(\mathrm{bpy}\right)}_{3}^{2+}$ decreased as the values of C_maxp_ and ECL_sl_ decreased. The magnitude of the effects of the input variables on the response variable can also be inferred from these plots. In this regard, it was observed that the concentration of $\mathrm{Ru}{\left(\mathrm{bpy}\right)}_{3}^{2+}$ was more sensitive to the variation of ECL_sl_ than C_maxp_. Contour plots were especially useful to display the system behavior, given the complexity of the developed models that are nonparametric, such as the RF, or that do not have simple prediction equations as the FNN. As in previous works [26,32], it can be noted that Figure 8a,b show typical behaviors of contour plots generated from a nonparametric model and a parametric model, respectively. In this study, the use of a reduced number of key features allowed for fast calibration and operation of the AI algorithms to predict the concentration of $\mathrm{Ru}{\left(\mathrm{bpy}\right)}_{3}^{2+}$. A greater number of key features could be considered in the construction of the data-driven models; however, some features could have a little or no effect on the response. Therefore, before incorporating more key features into the models, a sensitivity analysis should be performed to determine their potential contribution.

The use of the approach presented in this study to other applications, such as the detection of analytes of interest using the enhancing or quenching of their luminescent intensities, is straightforward. In this case, the concentration of $\mathrm{Ru}{\left(\mathrm{bpy}\right)}_{3}^{2+}$ must be fixed at an optimal value. For instance, phenolic compounds demonstrated a highly efficient quenching effect in the $\mathrm{Ru}{\left(\mathrm{bpy}\right)}_{3}^{2+}$/TPrA system [33]. In this sense, future work will take advantage of the results obtained in this study to develop an AI-driven smartphone-supported ECL sensor to monitor phenolic compounds in wastewater from biofuel plants. In this context, the present study is important because it provides a proof of concept demonstrating the feasibility to develop a sensor for intelligent detection of analytes.

## 4. Conclusions

The quantitative investigation of the relationships between the concentration of $\mathrm{Ru}{\left(\mathrm{bpy}\right)}_{3}^{2+}$ and its experimentally measured electrochemical and ECL features naturally leads to the use of complex models that are very difficult to calibrate. It is necessary to examine key features from the system to effectively consider the generalization of the model. This study proposes a novel modeling approach based on AI (in particular, random forest (RF) and feedforward neural network (FNN)) to correlate the concentration of $\mathrm{Ru}{\left(\mathrm{bpy}\right)}_{3}^{2+}$ with key features obtained from sequences of ECL imaging and amperograms. All multimodal measurements were extracted from a low-cost smartphone-based electrochemiluminescence (ECL) sensor. The input (key features) and output (concentration of $\mathrm{Ru}{\left(\mathrm{bpy}\right)}_{3}^{2+})$ variables were applied to generate sample points. These samples were used to build data-driven models using RFs and FNNs. The predictions of the data-driven models were shown to be in agreement with the measurements performed (validation and testing sets) with the mobile phone-based ECL sensor. Contour plots allowed quantitative determination of the relevance of the key features on the output and the relation between them. The AI approaches were capable of directly inferring the concentration of $\mathrm{Ru}{\left(\mathrm{bpy}\right)}_{3}^{2+}$ using easily observable key features, while traditional mechanistic modeling uses a complex calibration procedure. Future work will extend the proposed approach to develop a robust, practical, and affordable sensor for intelligent detection of analytes of economic relevance such as phenolic compounds.

## Figures and Tables

**Table 1:** Actual versus predicted values of the concentration of $\mathrm{Ru}{\left(\mathrm{bpy}\right)}_{3}^{2+}$ obtained for the testing set using the random forest and the feedforward neural network.

Testing Sample	Random Forest (RF) *R^2^* = 0.996, MSE = 0.0012		Feedforward Neural Network (FNN) *R^2^* = 0.961, MSE = 0.0356	
	$\mathrm{Concentration}\;\mathrm{of}\;\mathrm{Ru}{\left(\mathrm{bpy}\right)}_{3}^{2+}$		$\mathrm{Concentration}\;\mathrm{of}\;\mathrm{Ru}{\left(\mathrm{bpy}\right)}_{3}^{2+}$	
	Actual	Prediction	Actual	Prediction
1	1.25	1.253	0.156	0.185
2	1.25	1.304	2.5	2.472
3	0.078	0.105	1.25	0.926

**Table 2:** Optimized parameters (weights and bias) of the feedforward neural network.

	Parameters Connecting the Inputs and Hidden Neurons				Parameters Connecting the Hidden and Output Neuron	
	*w_j_*_1_	*w_j_*_2_	*w_j_*_3_	*θ_j_*	*W*_1*j*_	*b*_1_ = −0.46714
*j =* 1	−2.16914	0.54961	0.84096	0.96493	−0.11545	
*j =* 2	0.96444	−0.39983	0.54570	1.38495	−0.55877	
*j =* 3	−0.06212	0.76427	1.24634	−0.94330	−0.11051	
*j =* 4	−0.04506	5.42573	−1.99257	−0.36926	−0.16298	
*j =* 5	−1.42036	0.55738	−0.99856	−1.01188	1.50011	
*j =* 6	−1.65943	1.06460	−0.98453	−0.65498	1.92081	
*j =* 7	−2.57911	0.15109	−1.17164	2.19616	1.16145	
*j =* 8	−4.96551	−4.79277	0.00347	−0.31065	−2.83089	
*j =* 9	0.76280	−0.86469	−0.90831	0.40019	0.75119	
*j =* 10	1.10727	−0.04662	−0.60547	−0.14305	−1.12459	
*j =* 11	−2.98694	1.36294	−0.77255	0.09917	0.90778	
*j =* 12	1.04993	1.17599	−0.46819	0.39381	1.46889	
*j =* 13	−1.41821	−0.44610	1.58347	0.83625	−0.21712	
*j =* 14	1.22302	−5.44580	4.17545	0.97755	−0.45628	
*j =* 15	0.82019	−0.32754	0.59748	1.02389	−0.17525	
*j =* 16	2.46345	−1.47657	−2.04265	1.07287	0.69586	
